# The association among three aspects of physical fitness and metabolic syndrome in a Korean elderly population

**DOI:** 10.1186/s13098-015-0106-4

**Published:** 2015-12-12

**Authors:** Hee-Jin Hwang, Sang-Hwan Kim

**Affiliations:** Department of Family Medicine, International St. Mary’s Hospital, Catholic Kwandong University College of Medicine, Incheon, Republic of Korea; Institution for Translational and Clinical Research, International St. Mary’s Hospital, Catholic Kwandong University College of Medicine, Incheon, Republic of Korea; Yonsei Woori Geriatric Hospital, Goyang, Gyeonggi-do Republic of Korea

**Keywords:** Physical fitness, Metabolic syndrome, Cardiopulmonary fitness, Muscular endurance, Elderly

## Abstract

**Background:**

The purpose of current study is to evaluate the association between physical fitness level and the prevalence of metabolic syndrome (MS) in a Korean elderly population.

**Methods:**

A cross-sectional study measuring physical fitness and components of MS in a health promotion center of a general hospital for routine health check-ups. A total of 227 subjects >60 years old agreed to participate. A lifestyle questionnaire that included cigarette smoking, alcohol consumption, and physical activity was checked. Body weight, height, blood pressure, fasting glucose, total cholesterol, triglycerides, high density lipoprotein-cholesterol, low density lipoprotein-cholesterol, C-reactive protein, and glycated hemoglobin were measured. Muscular strength was assessed by measuring grasping power. Muscular endurance was evaluated using a sit-up test. Cardiopulmonary fitness was assessed via the Tecumseh step test (measuring hear rates at 1 min post-exercise).

**Results:**

The highest tertile muscular endurance group (sit-ups >15 in men, >10 in women) was 0.37 times less likely to have MS [95 % confidence interval (CI) 0.17–0.84; p = 0.036] compared to that in the lowest tertile group (<11 in men, 0 in women), and the highest tertile in the cardiopulmonary fitness group (heart rate at rest >91/min in men, >92/min in women) was 2.81 times more likely to have MS (95 % CI 1.27–6.18; p = 0.038) compared to that in the lowest tertile group (<81/min in men, <80/min in women).

**Conclusions:**

Cardiopulmonary fitness and muscular endurance were related to MS in a Korean elderly population after adjusting for age, sex, current smoker, current alcohol drinking, and physical activity. Older adults should be encouraged to increase their cardiopulmonary fitness and muscular endurance.

## Background

Metabolic syndrome (MS) is the term given to a group of interrelated risk factors of metabolic origin that appear to directly promote the development of cardiovascular diseases [1]. MS is strongly associated with type 2 diabetes mellitus [2]. The prevalence of MS are increasing rapidly and a study recently reported that the prevalence of MS is 22.1 % in Korean males and 27.8 % in Korean females [3]. Thus, preventing MS has become an important issue.

In general, people with MS have low physical performance, and high level physical fitness could be helpful to prevent the development of chronic diseases independent of adiposity in youth and adults [4, 5]. Physical fitness is defined as the ability to carry out work necessary for muscle exercise [6]. It is generally considered “the ability to perform daily tasks without fatigue” [7]. However, most studies that have demonstrated the relationship between physical fitness and MS were performed with young Caucasian adults, and relatively few studies have been conducted among the Asian elderly population. Therefore, the purpose of this study was to evaluate the association between physical fitness level and the prevalence of MS in a Korean elderly population.

## Methods

### Subjects

This cross-sectional study was explained to a Korean elderly population who visited a health promotion center of one general hospital in Gyeonggi province for routine health check-ups between November 2008 and February 2009, and 227 (121 men and 106 women) subjects >60 years old agreed to participate. Written informed consent was obtained from all subjects. This study was approved by the ethics review committee of a hospital.

### Measurements

#### Life style measurements

All subjects completed a lifestyle questionnaire that included questions about cigarette smoking, alcohol consumption, and physical activity. A smoking habit was defined as currently smoking cigarettes. Drinking more than 70 g/week was defined as alcohol drinking. Age and sex was checked by resident registration number. Current medical diseases such as diabetes mellitus and hypertension were collected from medical records. Subjects who had history of chronic liver disease or chronic renal disease were not included in this study.

#### Anthropometric measurements

Body weight (kg) and height (cm) were measured using JENIX automatic measuring equipment (Garden Jenix, Seoul, Korea) while subjects were wearing light-weight clothing or a hospital gown without shoes. Body mass index (BMI, kg/m^2^) was calculated as weight (kg) divided by height squared (m^2^), and waist circumference (WC, cm) was measured midway between the lowest rib and the iliac crest using a Gullick II tape with subjects in the standing position at the end of a normal expiration. All anthropometric measurements were performed by a single researcher.

#### Blood pressure (BP) and blood assay

BP was measured using a mercury sphygmomanometer (Hico, Tokyo, Japan) when the subjects were relaxed. All participants were asked to rest for 10 min in the sitting position prior to the measurement. Blood samples were collected for biochemical tests after overnight fasting (>12 h), and serum concentrations of fasting glucose, total cholesterol (TC), triglycerides (TG), high density lipoprotein-cholesterol (HDL-C), low density lipoprotein-cholesterol (LDL-C), and C-reactive protein were measured using an ADVIA 1650 Chemistry System (Siemens, Tarrytown, NY, USA). Glycated hemoglobin (HbA1C) was evaluated using a HLC-723GHb instrument (TOSOH, Siba, Minaoto-ku, Japan).

#### Physical fitness measurements

Muscular strength was assessed by measuring grasping power with a digital dynamometer (TKK 5101 Grip-D; Takey, Tokyo, Japan). The subject sat with shoulders adducted, elbows flexed, and forearms at the mid-position. The second joint was used to position the dynamometer, and the handle was adjusted according to finger length. The subject performed the test twice, allowing a 3 min rest period between measurements. The mean value of two trials was scored. This test is valid and reliable [8].

Muscular endurance was assessed using a sit-up test. The participants lay on sit-up equipment and performed sit-ups with their feet attached to the equipment’s foot holders. One sit-up was counted when the participants sat up so that their elbows touched their thighs, and they had returned to the supine position and both shoulders touched the equipment. The number of sit-ups performed in 30 s was recorded.

Cardiopulmonary fitness was assessed using the Tecumseh step test. Based on the guidelines for the Tecumseh step test, subjects performed 24 steps/min, maintaining a constant stepping rate on a 20.3 cm high step for 3 min. To help maintain a constant stepping rate, a metronome was used, and an assistant was present. The subjects wore HR monitors (FS3C; Polar Electro, Lake Success, NY, USA), and HRs were recorded prior to exercise and at each 1 min interval during the exercise session. HR was also measured for 1 min during the recovery phase. Participants with higher cardiopulmonary fitness would have lower HRs at 1 min post-exercise than those with lower cardiopulmonary fitness [9].

#### Physical activity (PA) assessment

PA level was evaluated by the International Physical Activity Questionnaire (IPAQ): short (last 7 days) self-administered format. The IPAQ has been used worldwide for PA surveillance, and an international validation has been reported previously [10]. In addition, the reliability and validity of the IPAQ Korean version has been established [11]. The Short IPAQ form is comprised of separate domain scores for walking, moderate-intensity and vigorous-intensity activities, and sedentary behavior, including work, transportation, domestic chores, gardening and leisure-time. Sedentary behavior (min/week) was time spent sitting during an ordinary weekday and weekend. Based on self-reported frequency (day) and duration (h) of physical activity, corresponding metabolic equivalent (MET-h/week) values were calculated using the following equations [12]:

$$ \begin{aligned} {\text{Walking }}\left( {{\text{MET}} {\text{-}} {\text{h}}/{\text{week}}} \right) \, = { 3}. 3\times {\text{walking hours}} \times {\text{walking days}} \hfill \\ {\text{Moderate PA }}\left( {{\text{MET}} {\text{-}} {\text{h}}/{\text{week}}} \right) \, = { 4}.0 \times {\text{moderate PA hours}} \times {\text{moderate PA days}} \hfill \\ {\text{Vigorous PA }}\left( {{\text{MET}} {\text{-}} {\text{h}}/{\text{week}}} \right) \, = { 8}.0 \times {\text{vigorous PA hours}} \times {\text{vigorous PA days}} \hfill \\ {\text{Total PA }}\left( {{\text{MET}} {\text{-}} {\text{h}}/{\text{week}}} \right) \, = {\text{ sum of walking }} + {\text{ moderate PA }} + {\text{ vigorous PA}} \hfill \\ \end{aligned} $$

#### Definition of MS

According to the National Cholesterol Education Program Adult Treatment Panel III (NCEP ATP III) standard, MS is diagnosed when a person has three or more components from the following list: abdominal obesity (WC >102 cm for males, >88 cm for females), high fasting glucose level (>100 mg/dL, fasting or taking diabetic medications), high blood pressure (systolic blood pressure >130 mm Hg or diastolic blood pressure >85 mm Hg or taking antihypertensives), high TG (>150 mg/dL), and low level of HDL-C (<40 mg/dL for males, <50 mg/dL for females) [1]. Due to a smaller body size, the Asian version of the modified NCEP ATP III definition was employed (WC: >90 cm in males, >80 cm in females) [13].

### Statistical analysis

The *t* test or Chi square test was used to compare differences between subjects with MS and subjects without MS. A non-normally distributed variable such as triglyceride was analyzed after log transformation. A partial correlation analysis was carried out to identify the relationship between physical fitness and components of MS. Each physical fitness item was categorized into tertiles to compute odds ratios (ORs) and 95 % confidence intervals (CIs) for MS. A logistic regression analysis was performed to assess the associations between physical fitness and MS after adjusting for age, sex, current smoker, current alcohol drinking, and physical activity. All statistical analyses were conducted using SPSS 17.0 (SPSS Inc., Chicago, IL, USA), and a p value <0.05 was considered significant.

## Results and discussion

Table 1 shows the characteristics of the subjects. Age, sex, smoking, drinking, diastolic blood pressure, total cholesterol, LDL-C, C-reactive protein, and physical activity were not different between subjects with MS and those without MS. BMI, WC, diabetes mellitus, hypertension, systolic blood pressure, fasting glucose, HbA1c, TG, and HDL-C were higher or more frequent in subjects with MS than those without MS.

**Table 1:** Baseline characteristics of the subjects according to metabolic syndrome (MS)

Characteristics	Without MS (n = 142)	With MS (n = 85)	P value
Age, years	66.9 ± 5.3	66.8 ± 5.3	0.879
Sex, male	79 (55.6)	43 (50.6)	0.461
Body mass index, kg/m^2^	23.5 ± 2.4	26.0 ± 2.6	<0.001
Waist circumference, cm	79.5 ± 7.0	86.3 ± 6.6	<0.001
Diabetes mellitus	10 (7.0)	14 (16.5)	0.025
Hypertension	49 (34.5)	46 (54.1)	0.004
Current smoker	45 (31.7)	29 (34.1)	0.706
Alcohol drinking	17 (12.0)	13 (15.3)	0.474
Systolic blood pressure, mmHg	126.6 ± 14.6	133.7 ± 12.3	<0.001
Diastolic blood pressure, mmHg	76.7 ± 9.8	79.1 ± 8.4	0.055
Fasting glucose,	98.9 ± 17.6	113.2 ± 23.6	<0.001
HbA1c, %	5.80 ± 0.72	6.12 ± 0.72	<0.001
Total cholesterol, mg/dL	193.3 ± 32.6	192.6 ± 35.3	0.885
Triglyceride^a^, mg/dL	109.2 ± 50.4	176.9 ± 92.1	<0.001
HDL-cholesterol, mg/dL	52.1 ± 12.5	42.6 ± 9.4	<0.001
LDL-cholesterol, mg/dL	120.4 ± 28.4	123.0 ± 32.1	0.513
C-reactive protein^a^, mg/dL	0.18 ± 0.41	0.18 ± 0.29	0.054
Total Physical activity^a^, METs	2192.2 ± 3130.8	1884.1 ± 1980.2	0.294
Low	84 (59.2)	45 (52.9)	
Moderate	43 (30.3)	31 (36.5)	0.611
Vigorous	15 (10.6)	9 (10.6)	

Data are shown as mean ± standard deviation or number (%). P values were calculated by the *t*-test or Chi square test

^a^Values were analyzed after log transformation

BMI and WC were positively associated with grasping power. TG was negatively associated with the number of sit-ups on the sit-up test. Diastolic blood pressure and fasting glucose were positively associated with HR at rest (Table 2).

**Table 2:** Partial correlation analysis between physical fitness and the components of metabolic syndrome

	Grasping power		Sit-up		HR 1 min post exercise	
	r	P value	r	P value	r	P value
BMI	0.200	0.003	0.103	0.123	0.055	0.410
Waist circumference	0.136	0.041	−0.005	0.938	0.111	0.095
Systolic blood pressure	0.050	0.454	−0.060	0.369	0.091	0.175
Diastolic blood pressure	0.056	0.405	−0.016	0.807	0.169	0.011
Fating glucose	0.072	0.282	−0.098	0.142	0.178	0.007
Log (triglycerides)	−0.050	0.460	−0.199	0.003	0.023	0.736
HDL-cholesterol	−0.015	0.825	0.091	0.174	−0.030	0.649
Grasping power			0.116	0.084	−0.001	0.989
Sit-up	0.116	0.084			−0.028	0.680
HR 1 min post exercise	−0.001	0.989	−0.028	0.680		

Coefficients (r) and P values were calculated by the partial correlation analysis after adjusting for age and sex

The associations between each physical fitness item as tertiles and the risk of MS are reported in Table 3. In multivariate models adjusted for age, sex, current smoker, current alcohol drinking, and physical activity, the number of sit-ups was negatively associated with MS risk (highest vs. lowest tertile OR, 0.37; 95 % CI 0.17–0.84) and subjects in the third tertile (highest) of HR at rest were about three times more likely to have MS compared to subjects in the first tertile (OR, 2.81; 95 % CI 1.27–6.18).

**Table 3:** Odd ratios for metabolic syndrome according to physical fitness

	Q1	Q2	Q3	p for trend
Grasping power				
Unadjusted	1	1.44 (0.75–2.79)	1.50 (0.76–2.94)	0.431
Model 1	1	1.44 (0.74–2.82)	1.51 (0.75–3.03)	0.446
Model 2	1	1.21 (0.59–2.49)	0.92 (0.43–1.97)	0.755
Model 3	1	1.19 (0.56–2.51)	0.91 (0.41–2.03)	0.778
Sit-up				
Unadjusted	1	0.65 (0.34–1.24)	0.65 (0.33–1.27)	0.334
Model 1	1	0.65 (0.34–1.25)	0.65 (0.33–1.29)	0.346
Model 2	1	0.43 (0.20–0.91)	0.36 (0.16–0.78)	0.023
Model 3	1	0.44 (0.20–0.95)	0.37 (0.17–0.84)	0.036
HR 1 min post exercise				
Unadjusted	1	1.73 (0.87–3.44)	2.59 (1.31–5.12)	0.024
Model 1	1	1.72 (0.86–3.44)	2.58 (1.30–5.12)	0.025
Model 2	1	1.74 (0.81–3.76)	2.58 (1.21–5.53)	0.050
Model 3	1	1.81 (0.82–4.01)	2.81 (1.27–6.18)	0.038

Model 1: adjusted for age and sex

Model 2: adjusted for body mass index

Model 3: adjusted for age, sex, current smoker, current alcohol drinking, and physical activity

Grasping power: quartile (Q)1 (<32 kg in men, <19 kg in women), Q2 (32–37 kg in men, 19–23 kg in women), Q3 (≥37 kg in men, ≥23 kg in women)

Sit-ups: Q1 (<11 in men, 0 in women), Q2 (11–15 in men, 1–10 in women), Q3 (≥15 in men, ≥ 10 in women)

HR 1 min post exercise: Q1 (<81/min in men, <80/min in women), Q2 (81–91/min in men, 80–92/min in women), Q3 (≥91/min in men, ≥92/min in women)

This study reported the the association of physical fitness and prevalence of metabolic syndrome in selected population, in other words, elderly subjects and Korean population with measuring complete physical fitness.

MS is very prevalent in many countries worldwide. Many cross-sectional studies support an inverse association between physical activity and MS, but few studies have been conducted on the association between physical fitness and MS. Furthermore, most of those studies focused on cardiopulmonary fitness using a cycle ergometer or treadmill test. The present study checked physical fitness from three aspects: muscular strength measuring grasping power, muscular endurance using a sit-up test, and cardiopulmonary fitness by the Tecumseh step test.

BMI, WC, diabetes mellitus, hypertension, fasting glucose, HbA1c, and TG were higher or more frequent in the MS group, whereas HDL-C was lower, as these parameters are included in the diagnostic criteria of MS. TC, LDL-C, and physical activity were not different between subjects with MS and those without MS. Physical activity levels were not different between the MS and non-MS groups.

Previous studies [14–20] on cross-sectional associations between physical fitness and MS have reported a significant inverse association between fitness and the prevalence of MS. Carnethon et al. reported low fitness (<20th percentile) were three- to sixfold more likely to develop diabetes, hypertension, and the metabolic syndrome than participants with high fitness (≥60th percentile) [14]. In Quebec family study, fitness was negatively associated with most individual components of the metabolic syndrome, except HDL-cholesterol [15]. Prevalence of the MS was markedly lower across progressively higher levels of cardiorespiratory fitness(CRF) [16]. A dose–response association was present between the number of components of the MS and exercise capacity [17]. individuals with lowest CRF compared to those with highest CRF had 3.1- and 11.8-fold higher risk of having 2 and ≥3 MS components, respectively [18]. All of these studies checked cardiopulmonary fitness via a treadmill test or cycle ergometer as a measure of physical fitness. However, these methods require time, qualified personnel, and resources. The Tecumseh step test used in the current study is a relatively quick and easy test for measuring cardiopulmonary fitness and the correlation between the results of the 3 min step test and maximum oxygen consumption has been validated [9]. In the present study, cardiopulmonary fitness assessed by the Tecumseh step test was negatively associated with MS. Cardiopulmonary fitness is the ability to exercise continuously for extended periods without tiring while working the heart and lungs. Increased cardiopulmonary fitness is known to reduce the risk of cardio-metabolic diseases [19–23]. Low cardiopulmonary fitness is an independent predictor of all-cause and cardiovascular disease mortality. Cardiopulmonary fitness is considered to affect these comorbidities by regulating cardiac output and blood pressure [24].

The strength of the present study is that we checked two additional aspects of physical fitness such as muscular strength and muscular endurance. Grasping power is proportional to skeletal muscle mass, which could be related to body mass in general and could explain the positive correlation between grasping power and BMI and WC. Although muscular strength was not associated with MS in the present study, another study showed that lower grip strength was significantly associated with increased odds of having MS [25]. The number of sit ups on the sit-up test was positively correlated with fasting glucose. Insulin resistance is thought to be associated with central obesity. The relationship between the sit-up test and MS could be due to central adiposity [26]. Thus, measures of muscular endurance could be a marker for the risk of MS.

Several limitations of the present study should be recognized. First, the cross-sectional nature of this study precluded our ability to identify any cause-effect relationships. Second, self-reported physical activity is almost always substantially higher than that measured objectively, and, therefore, it might lead to an attenuation of the effect of physical activity on MS in the elderly. Third, the prevalence of MS in this study (36 % for male and 40 % for female) were much higher than those of MS in national representative data (22.1 % in Korean males and 27.8 % for females) 0.3 The discrepancy could be due to the fact that the study sample used in the current study was older adults. Also, there could be a selection bias since the study sample was selected from only one general hospital in Gyeonggi province, the most populous province of Korea, although we enrolled all elderly who who visited for routine health check-ups and agreed to participate. Fourth, muscular strength is validated in the adolescent, not the older adults.

## Conclusions

Cardiopulmonary fitness and muscular endurance were related to MS in a Korean elderly population after adjusting for age, sex, current smoker, current alcohol drinking and physical activity. In conclusion, not only cardiopulmonary fitness but also strengthening muscle should be recommended in practice, services or health care policy, especially for elderly.
